# Effect of Degeneration on Fluid–Solid Interaction within Intervertebral Disk Under Cyclic Loading – A Meta-Model Analysis of Finite Element Simulations

**DOI:** 10.3389/fbioe.2015.00004

**Published:** 2015-01-28

**Authors:** Mohammad Nikkhoo, Kinda Khalaf, Ya-Wen Kuo, Yu-Chun Hsu, Mohammad Haghpanahi, Mohamad Parnianpour, Jaw-Lin Wang

**Affiliations:** ^1^Department of Biomedical Engineering, Science and Research Branch, Islamic Azad University, Tehran, Iran; ^2^Institute of Biomedical Engineering, College of Medicine and Engineering, National Taiwan University, Taipei, Taiwan; ^3^Department of Biomedical Engineering, Khalifa University of Science, Technology and Research, Abu Dhabi, UAE; ^4^School of Mechanical Engineering, Iran University of Science and Technology, Tehran, Iran; ^5^Department of Industrial and Manufacturing, University of Wisconsin, Milwaukee, WI, USA

**Keywords:** poroelastic FE model, porcine intervertebral disk, fluid–solid interaction, response surface methodology, artificial degeneration

## Abstract

The risk of low back pain resulted from cyclic loadings is greater than that resulted from prolonged static postures. Disk degeneration results in degradation of disk solid structures and decrease of water contents, which is caused by activation of matrix digestive enzymes. The mechanical responses resulted from internal solid–fluid interactions of degenerative disks to cyclic loadings are not well studied yet. The fluid–solid interactions in disks can be evaluated by mathematical models, especially the poroelastic finite element (FE) models. We developed a robust disk poroelastic FE model to analyze the effect of degeneration on solid–fluid interactions within disk subjected to cyclic loadings at different loading frequencies. A backward analysis combined with *in vitro* experiments was used to find the elastic modulus and hydraulic permeability of intact and enzyme-induced degenerated porcine disks. The results showed that the averaged peak-to-peak disk deformations during the *in vitro* cyclic tests were well fitted with limited FE simulations and a quadratic response surface regression for both disk groups. The results showed that higher loading frequency increased the intradiscal pressure, decreased the total fluid loss, and slightly increased the maximum axial stress within solid matrix. Enzyme-induced degeneration decreased the intradiscal pressure and total fluid loss, and barely changed the maximum axial stress within solid matrix. The increase of intradiscal pressure and total fluid loss with loading frequency was less sensitive after the frequency elevated to 0.1 Hz for the enzyme-induced degenerated disk. Based on this study, it is found that enzyme-induced degeneration decreases energy attenuation capability of disk, but less change the strength of disk.

## Introduction

Excessive cyclic loading requested by occupations in daily lives, such as repetitive lifting and vibration working environments, is recognized as a risk factor of low back pain (Marras et al., 1995). The risk of low back pain resulted from cyclic loadings is three times greater than the one resulted from prolonged static postures (Bigos et al., 1986). The mechanics of low back is a very complex process but it assumes that the failure can be observed primarily at the vertebral bodies, intervertebral disks, and endplates because of axial compressive cyclic loading (Liu et al., 1983; Marras et al., 2006; Wang et al., 2008; Stefanakis et al., 2014). The failure can also cause disk protrusion due to excessive tasks during life. The underlying reason that cyclic loading induces low back pain could be the micro injuries of disk solid structures, which leads to water over exudation (Qasim et al., 2014).

Intervertebral disk is a non-linearly permeable, viscoelastic material consisting of two main phases, i.e., a charged solid phase and a fluid phase. The solid phase is composed of a strong collagen fibrillar network enmeshed with a high concentration of charged proteoglycan and the fluid phase is water (Mow et al., 1999). The interactions of disk solid structures (i.e., collagen fibril network, proteoglycan) and interstitial fluid determines disk endurance to cyclic loadings. Disk degeneration results in degradation of solid structures and loss of water contents (Le Maitre et al., 2007). As a result, the pattern of interactions between disk solid structures and interstitial fluid is altered, making disks more vulnerable to cyclic loading (Inkinen et al., 1998; Wang et al., 2008). However, how this fluid–solid interaction pattern changes with cyclic loading has not been well studied yet.

Animal models can be used to mimic disk degeneration. The methods used to create artificial disk degeneration include the injection of trypsin, chondroitinase ABC (ChABC), stab injury, and mechanical loading in animal models (Kroeber et al., 2002; Norcross et al., 2003; Roberts et al., 2008; Chuang et al., 2010; Hsu et al., 2013; Paul et al., 2013; Mwale et al., 2014). These models are used to simulate different grades of disk degeneration. Injection of the trypsin makes the disturbance of proteoglycans and loosens the extracellular matrices. Former studies showed that an interrupted transition between the anulus fibrosus and nucleus pulposus, a muddled anular fibrils, a condensation of the extracellular matrix of the nucleus pulposus, and an increase of nucleus cell density presented in trypsin-induced degenerated disks are similar to those seen in mild disk degeneration (Mao et al., 2011; Zhang et al., 2011; Hsu et al., 2013; Gawri et al., 2014).

The fluid–solid interactions in disks can be evaluated by mathematical models, especially the one based on poroelastic theory (Simon et al., 1985; Argoubi and Shirazi-Adl, 1996; Williams et al., 2007; Schmidt et al., 2010; Castro et al., 2014). A poroelastic finite element (FE) model can be used to observe the fluid transferring, disk stress deviation, and other properties. Among parameters of a FE model, hydraulic permeability (*k*) and elastic modulus (*E*) regulate fluid outflow and disk strength, respectively. The choices of values of these two parameters affect the robustness of model predictions on fluid–solid interactions in disks during cyclic loadings. Understanding the effects of these parameters on time-dependent response of intervertebral disk can be beneficial to enhance understanding of changes in material properties during disk degeneration. The purpose of this study was to develop a robust disk poroelastic FE model to analyze the effect of degeneration on solid–fluid interactions within disk subjected to cyclic loadings at different loading frequencies.

## Materials and Methods

### *In vitro* experiment

A total of 18 porcine thoracic motion segments were dissected from 6-month-old juvenile pigs within 4 h after death. Each specimen was carefully removed of muscle, posterior elements, and facet joints. The upper and lower vertebral bodies were cut parallel to warrant pure axial compressive deformation. The initial disk height and dimensions were measured using digital caliper. Specimens were wrapped in phosphate buffer saline (PBS) soaked gauze, sealed in plastic bag, and stored at −20°C in a refrigerator until experiment.

Specimens were equally assigned to intact and degeneration groups. The disk of intact group did not receive any forms of injury. Disk degeneration was simulated by injecting 0.5 ml trypsin solution (0.5%) into nucleus pulposus. Specimens were mounted in center of a home-made chamber filled with PBS solution to mimic the physiological environment of spine. A Teflon plate was attached to the actuator of the material testing machine to transfer cyclic loadings to the specimen. After preloading (0.1 MPa compression, 10 min), a sinusoid cyclic load (Peak-to-peak, 0.1-to-0.8 MPa) was applied to the specimens with frequencies sweeping at 0.01, 0.063, 0.1, 0.63, 1, 6.3, and 10 Hz by a material testing apparatus (ElectroForce^®^ 3510, Bose Corporation, MN, USA).

After each mechanical test, specimens were cut into two parts from the midline. One half of the disk was assigned for measuring the water content and the other half was used for histological analyses. Before assessing the regional water contents for the nucleus pulposus, inner anulus fibrosus, and outer anulus fibrosus, the tissue samples were weighed and dried by lyophilization technique.

### Poroelastic FE model establishment

**Figure 1:**
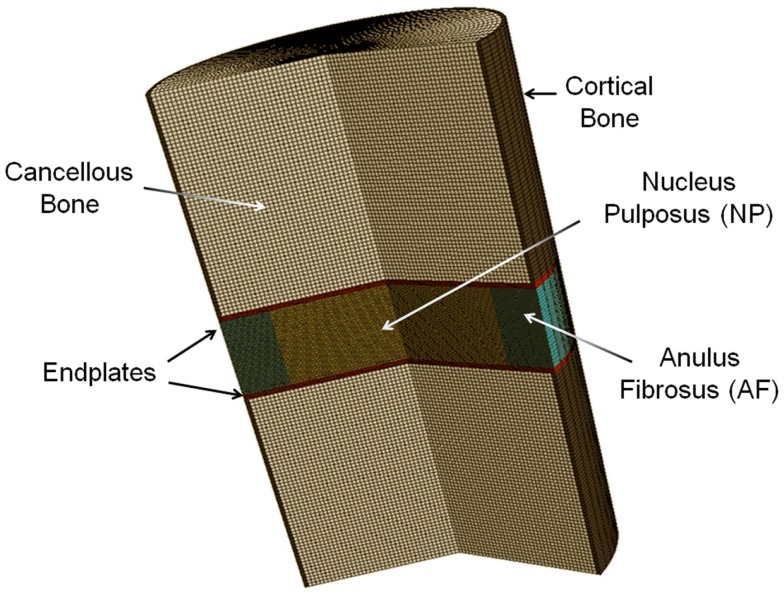
**Revolved axisymmetric poroelastic FE model of porcine intervertebral disk**.

Based on porous media theory, a general poroelastic FE model of intervertebral disks was developed using ABAQUS v6.9 (SIMULIA, Providence, RI, USA) (Figure 1). In this model, the solid matrix deforms and the fluid flows within the solid matrix. The fluid flows from higher pressure to lower pressure changes the stress and strain field of the matrix. The friction between solid and fluid makes the behavior of the material to be rate-dependent.

This model was previously validated (Nikkhoo et al., 2013a) by *in vitro* human disk experiments for both creep (Heuer et al., 2007) and cyclic loading (Li, 1994). With the aim of using the model for investigation of the porcine tests, the geometry and initial conditions of water content were modified for intact and enzyme-induced degenerative porcine disk. For this purpose, the radii, disk heights, and porosities of 18 simulated disks were measured with the individual tested disks (Table 1). The porosity was calculated based on the density and measured water content of the disk (Hillel, 2003). The density of the anulus fibrosus and nucleus pulposus were assumed to be 1.06 and 1.0 g/cm^3^, respectively (Williams et al., 2007). Eight-node axisymmetric elements with quadratic interpolation of displacement field and linear interpolation of pore pressure were used. The numbers of nodes and elements were 19916 and 6517, respectively. Non-linear large deformations were used for calculation. To ensure the convergence, the maximum load was divided into four substeps, whereas each substep was iteratively determined using the Newton’s method. The mesh density was shown to be sufficient by a sensitivity analysis (Nikkhoo et al., 2011, 2013c).

**Table 1:** **Average disk radius, height, and porosity of intact and enzyme-induced degenerative porcine intervertebral disks (*N* = 18)**.

Group	Intact disk	Enzyme-induced degenerated disk	*p* value
Disk radius (mm)^a^	13.18 (0.32)	13.29 (0.27)	0.442
Disk height (mm)	4.21 (0.34)	2.95 (0.28)	<0.001*
Porosity of anulus fibrosus	0.80 (0.03)	0.78 (0.04)	0.248
Porosity of nucleus pulposus	0.92 (0.04)	0.86 (0.05)	0.013*

*^a^The radius of the disk was assumed to be the geometric average of the long and short axes of the porcine disk*.

*Statistical differences were considered to be significant at *p* < 0.05.

The inferior surface of the lower vertebra was fixed. The axial displacement of the superior surface of upper vertebra was assumed to be consistent. The fluid was free to flow and no element was allowed to slide along the interface of different materials. The swelling phenomenon was simulated by imposing a fixed boundary pore pressure (Galbusera et al., 2011) on the external surfaces of intervertebral disk. Based on a sensitivity analysis, the boundary pore pressure of 0.3 MPa was chosen as it yielded the best agreement for the reduction of both disk height loss and pore pressure in validation procedure (Nikkhoo et al., 2013a). A linear ramp from 0 to 0.1 MPa over 60 s followed by a 10-min unconfined loading was applied on the superior surface of upper vertebra as preloading. Furthermore, a sinusoid cyclic load (Peak-to-peak, 0.1-to-0.8 MPa) was applied on the superior surface of upper vertebra with different frequencies sweeping at 0.01, 0.063, 0.1, 0.63, 1, 6.3 in 300 s.

### Calculation of IVD mechanical properties

A validated backward analysis algorithm and quadratic response surface (QRS) regression (Nikkhoo et al., 2013c) were applied to find the optimal sets of mechanical properties for intact and enzyme-induced degenerated disk. This validated algorithm correlated the peak-to-peak disk displacement during cyclic loading experiments to the ones obtained from poroelastic FE simulations. For this purpose, two main material properties, elastic modulus (*E*) and hydraulic permeability (*k*), were selected as the independent variables of the FE model. To simplify the optimization procedure, the ratio of the elastic modulus for the anulus fibrosus and nucleus pulposus was assumed to be 1.67 and the ratio of hydraulic permeability were assumed to be 1 during analysis (i.e., *E*_AF_ = 1.67*E*_NP_, *k*_AF_ = *k*_NP_) (Argoubi and Shirazi-Adl, 1996; Schmidt et al., 2010).

A full factorial design of experiment (DOE) for the FE simulation was conducted using MATLAB (Mathworks, Inc., Natick, MA, USA). Two factors, i.e., *E* and *k*, and 5 levels in each factor resulted in 25 input combinations sets. Based on our previous studies (Nikkhoo et al., 2013b,c), the initial ranges of *E* for anulus fibrosus and nucleus pulposus were set from 2 to 3 MPa, and from 1.2 to 1.8 MPa, for both intact and enzyme-induced degenerated disk. As well, the ranges of *k* were set from 1.56 to 2.34 × 10^−16^ m^4^/Ns for the intact disk and from 1.18 to 1.76 × 10^−16^ m^4^/Ns for the enzyme-induced degenerated disk. Twenty-five cyclic loading simulations were performed for each specimen. To assess the correlation between the experimental and FE-simulated disk deformations, root mean square (RMS) error was calculated. With 25 sets of RMS errors and input parameters (*E*/*k*), QRS model was constructed and optimized to find the best set of *k*/*E*. The results were considered to be accurate when the RMS error was <5%. If it was larger than 5%, the input ranges of independent variables were modified to spread the searching window.

### Statistical analysis

Based on the results of the *in vitro* experiments and FE backward analysis, elastic modulus and hydraulic permeability of the 18 intact and enzyme-induced degenerated disks were calculated. The differences of the elastic modulus and hydraulic permeability between intact and enzyme-induced degenerated disks were evaluated using the independent *t*-test (SPSS, SPSS Inc., Chicago, IL, USA). Statistical differences were considered to be significant at *p* < 0.05. To evaluate how well the elastic modulus and hydraulic permeability can differentiate intact disks from enzyme-induced degenerated ones, a two-group linear discriminant analysis (LDA) was performed.

### FE analysis based on updated models

Based on the updated poroelastic FE models, intradiscal pressure, total fluid loss, and axial stress within solid matrix of intact and enzyme-induced degenerated intervertebral disk under four types of cyclic loadings were investigated. For these simulations, the boundary conditions were considered the same as the described model in Section “Poroelastic FE Model Establishment.” A linear ramp from 0 to 0.1 MPa over 60 s followed by a 10-min unconfined loading was applied on the superior surface of upper vertebra as preloading. Furthermore, a sinusoid cyclic load was applied on the superior surface of upper vertebra. For this purpose, periodic sinusoid amplitude was defined. The peak-to-peak pressure of each cyclic loading was 0.1-to-0.8 MPa and different loading frequencies were 0.01, 0.1, 1, and 10 Hz, respectively. The simulations were performed for 1 h which means 36, 360, 3600, and 36,000 cycles and increment times were chosen 5, 0.5, 0.05, and 0.005 s, respectively.

## Results

**Figure 2:**
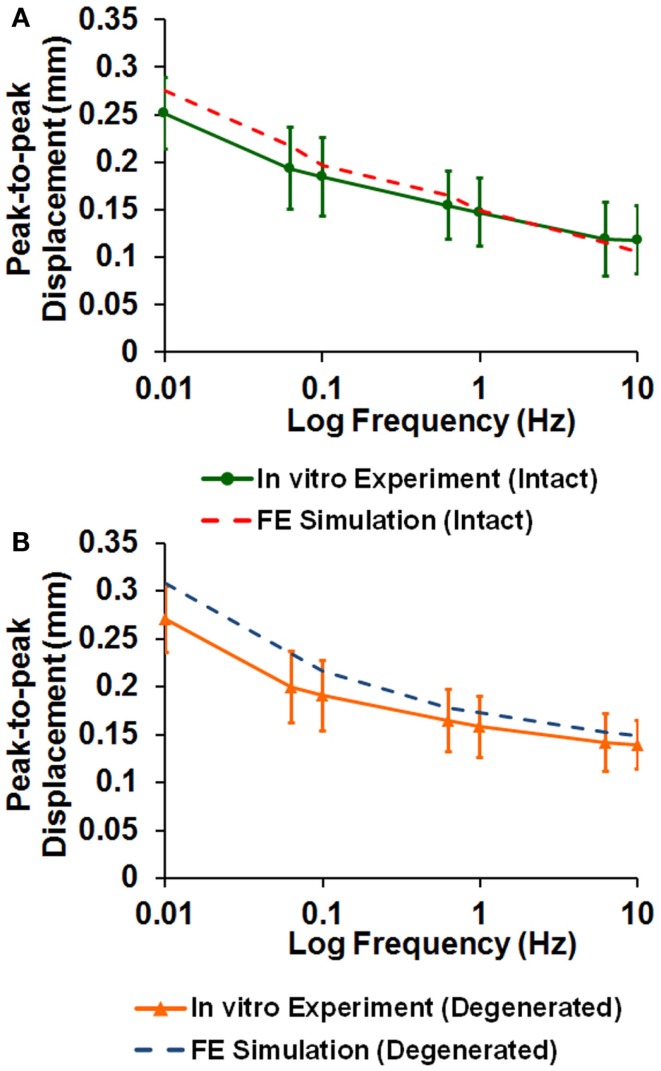
**Comparison of disk deformation obtained from *in vitro* cyclic loading experiments and the ones predicted by poroelastic FE model for (A) intact and (B) enzyme-induced degenerative intervertebral disks**.

The averaged peak-to-peak disk deformations during cyclic loading experiments were well fitted by FE model predictions for both intact (Figure 2A) and enzyme-induced degenerated disks (Figure 2B). The average normalized percentages of RMS error were 3.84 (0.96) and 3.42 (0.79)% for the intact and enzyme-induced degenerated disks, respectively. For the intact disks, the average elastic modulus of anulus fibrosus and nucleus pulposus were 2.35 and 1.41 MPa, and the average hydraulic permeability was 2.17 × 10^−16^ m^4^/Ns (Table 2). For the enzyme-induced degenerated disk, the average elastic modulus of anulus fibrosus and nucleus pulposus decreased to 2.16 and 1.30 MPa, and the average hydraulic permeability decreased to 1.39 × 10^−16^ m^4^/Ns (Table 2). The results of discriminate analysis showed that 88.9% of intact disks (8 of 9 intact disks) correctly classified as intact group using the current backward material property identification algorithm. This criterion was 77.8% (7 of 9 enzyme-induced degenerated disks) for enzyme-induced degenerated disks group.

**Table 2:** **Comparison of elastic modulus and hydraulic permeability between intact and enzyme-induced degenerative porcine intervertebral disks**.

Group	Intact disk	Enzyme-induced degenerated disk	*p* value
Elastic modulus (AF) (MPa)	2.35 (0.31)	2.16 (0.23)	0.159
Elastic modulus (NP) (MPa)	1.41 (0.17)	1.30 (0.14)	0.110
Hydraulic permeability (AF and NP) (m^4^/Ns)	2.17 (0.36) × 10^−16^	1.39 (0.29) × 10^−16^	<0.001*

*Statistical differences were considered to be significant at *p* < 0.05.

**Figure 3:**
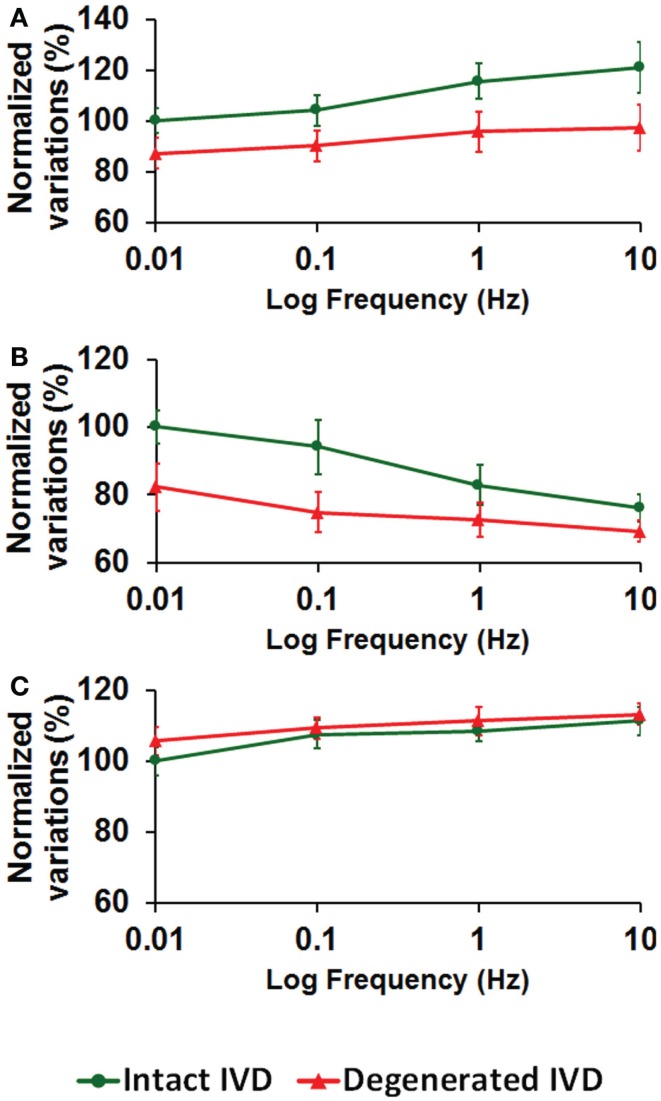
**The effect of cyclic loading frequency on the (A) intradiscal pressure (B) total fluid loss (C) axial stress for intact and enzyme-induced degenerative intervertebral disks (the results were normalized to the response of intact disk at 0.01 Hz)**.

Higher loading frequency increased the intradiscal pressure (Figure 3A), decreased the total fluid loss (Figure 3B), and slightly increased the maximum axial stress within solid matrix (Figure 3C). Enzyme-induced degeneration decreased the intradiscal pressure and total fluid loss (Figures 3A,B), and barely changed the maximum axial stress within solid matrix (Figure 3C). For the enzyme-induced degenerated disk, the increase of intradiscal pressure and total fluid loss with loading frequency was less sensitive after the frequency elevated to 0.1 Hz (Figures 3A,B).

## Discussion

This study presented a meta-analysis of *in vitro* porcine disk model using a poroelastic FE model to find the effect of cyclic loading frequency on the fluid–solid interaction in intact and enzyme-induced degenerated disks. To investigate the time-dependent response of intervertebral disk, an *in vitro* porcine disk model was used which is comparable to the human disk in the aspect of histology, geometry, and the mechanical properties (Mclain et al., 2002; Beckstein et al., 2008). In this study, the disks were treated with trypsin to mimic the disk degeneration. Injection of the trypsin makes the disturbance of proteoglycans and loosens the extracellular matrices. Based on the methodology proposed by Zhang et al. (2011) and Pfirrmann et al. (2001), our histological study showed that enzyme-induced degenerated disk would be comparable with the grade I degeneration of the magnetic resonance imaging grading system.

It was found that intradiscal pressure and fluid loss are more sensitive to cyclic loading frequency compared to axial stress. Increase of loading frequency limits the total fluid loss of disk, and thus increases the intradiscal pressure and axial stress of the solid matrix. Intradiscal pressure and fluid loss are mainly related to the hydraulic permeability and porosity of the intervertebral disk. The hydraulic permeability describes the fluid flow capability within disk matrix. It has a higher sensitivity on the saturation time for disk deformation, which means that the hydraulic permeability would regulate the fluid flow within the tissue matrix and the convergence to the equilibrium (Nikkhoo et al., 2013a). The axial stress varies regarding to the stiffness of disk matrix, which is not sensitive to the loading frequency.

The magnitudes of hydraulic permeability of enzyme-induced degenerated disk are significantly lower than of intact one. It is supposed that the injected digestive agent, i.e., trypsin, disrupts the functional interactions of proteoglycans. The collapse of disk structure interferes with fluid outflow, which is reflected in lower hydraulic permeability. Therefore, enzyme-induced degeneration decreases intradiscal pressure and hinders the fluid outflow (indicated by total fluid loss). Reduction of intradiscal pressure and fluid outflow reduces energy absorption capability of disk. As well, the degraded proteoglycan also decrease the water content and damping coefficient (Hsu et al., 2013). This is consistent with earlier studies that extracellular degradation directly impacts the mechanical function of the intervertebral disk (Iatridis et al., 1998; Johannessen and Elliott, 2005; Perie et al., 2006). The denaturation of proteins occurs in the early stage of disk degeneration, and leads to a change in the biomechanical property and pressure redistribution of the intervertebral disk (Schollum et al., 2010).

Few limitations of this study should be addressed. First, the geometry of the FE model was axisymmetric and the posterior elements and facet joints were removed. In this study, only the axial cyclic loading was applied and displacement response was investigated. Hence, the 2D axisymmetric model was sufficient for the optimization procedure. Second, the ratio of elastic modulus and hydraulic permeability of anulus fibrosus and nucleus pulposus was assumed to be constant (Argoubi and Shirazi-Adl, 1996; Schmidt et al., 2010) in our calculations. This simplification was considered to reduce the number of independent variables. If different ratio of elastic modulus and hydraulic permeability for anulus fibrosus and nucleus pulposus were allowed, 4 independent variables were needed which rise the number of simulations from 25 to 625 and increases the run time for constructing a QRS model. Nonetheless, degeneration could change the ratio of material properties between the anulus fibrosus and nucleus pulposus that was neglected with this simplification. Third, due to the complexity of anisotropic characteristics, the poroelastic FE model was considered to be isotropic. This simplification reduced the number of simulations from 15625 to 25 and made it feasible. In practice, most FE intervertebral disk models (Argoubi and Shirazi-Adl, 1996; Schmidt et al., 2010; Galbusera et al., 2011) use the isotropic poroelastic material properties for annulus ground substance, which is reinforced with truss or rebar elements in different layers. The crisscross structured reinforced anulus fibers within the anulus matrix were also not considered in this study. This is approximately justified due to the negligible effects of the fibers under pure axial compression loads. Therefore, the elastic modulus obtained for both intact and enzyme-induced degenerated disks in this study represents the overall stiffness of the anulus fibrosus. Fourth, the swelling phenomenon was simulated by imposing a same value of fixed boundary pore pressure (0.3 MPa) on the external surfaces of both intact and the enzyme-induced degenerated disks. Actually, enzyme-induced degenerated disks may show lower swelling due to the reduced proteoglycan content but the measurement of individual fixed boundary pore pressure for simulations were not possible in our experiments. To clarify the influence of this value in optimization procedure, simulations for some intact and enzyme-induced degenerated disk models repeated for three different values of boundary pore pressure (i.e., 0.3, 0.25, and 0.2 MPa). The results showed that the variation of the elastic modulus and permeability for both intact and enzyme-induced degenerated disks with different values of boundary pore pressure were <5%. This sensitivity analysis showed that boundary pore pressure does not show significant effect on calculated independent parameters in cyclic loading and it is not that crucial. Hence, we included the same boundary pore pressure in the model to simulate the swelling phenomenon and represent its mechanistic contribution. However, we predicted to develop our calculations for future studies using 3D model with UMAT subroutine.

In conclusion, this study combined FE simulations, *in vitro* experiments, and QRS regression to provide a methodology to investigate the effect of cyclic loading frequency and disk degeneration on fluid–solid interaction of disk. The novelty of this study was to analyze the effect of degeneration on solid–fluid interactions within disk subjected to cyclic loadings at different loading frequencies. The safe limits of repetitive activities and whole body vibration that avoid spinal disease would be beneficial in understanding the potentially detrimental effects of exposure to loading. During the cyclic loading, the fluid of disk is squeezed out. Reduction of fluid loss would inhibit the nutrition transport and metabolism process within the disk, hence forms a vicious circle for the disk. The results of this study may be valuable to enhance understanding of changes in time-dependent response of intact and moderate enzyme-induced degenerated disk during repetitive activities. Based on this method, it is found that enzyme-induced degeneration decreases energy attenuation capability of disk, but less change the strength of disk.

## Conflict of Interest Statement

The authors declare that the research was conducted in the absence of any commercial or financial relationships that could be construed as a potential conflict of interest.
